# Patient and Public Willingness to Share Personal Health Data for Third-Party or Secondary Uses: Systematic Review

**DOI:** 10.2196/50421

**Published:** 2024-03-05

**Authors:** Rebecca Baines, Sebastian Stevens, Daniela Austin, Krithika Anil, Hannah Bradwell, Leonie Cooper, Inocencio Daniel Maramba, Arunangsu Chatterjee, Simon Leigh

**Affiliations:** 1 Centre for Health Technology University of Plymouth Plymouth United Kingdom; 2 Prometheus Health Technologies Ltd Newquay United Kingdom; 3 University of Plymouth Plymouth United Kingdom; 4 School of Medicine University of Leeds Leeds United Kingdom; 5 Warwick Medical School University of Warwick Conventry United Kingdom

**Keywords:** data sharing, personal health data, patient, public attitudes, systematic review, secondary use, third party, willingness to share, data privacy and security

## Abstract

**Background:**

International advances in information communication, eHealth, and other digital health technologies have led to significant expansions in the collection and analysis of personal health data. However, following a series of high-profile data sharing scandals and the emergence of COVID-19, critical exploration of public willingness to share personal health data remains limited, particularly for third-party or secondary uses.

**Objective:**

This systematic review aims to explore factors that affect public willingness to share personal health data for third-party or secondary uses.

**Methods:**

A systematic search of 6 databases (MEDLINE, Embase, PsycINFO, CINAHL, Scopus, and SocINDEX) was conducted with review findings analyzed using inductive-thematic analysis and synthesized using a narrative approach.

**Results:**

Of the 13,949 papers identified, 135 were included. Factors most commonly identified as a barrier to data sharing from a public perspective included data privacy, security, and management concerns. Other factors found to influence willingness to share personal health data included the type of data being collected (ie, perceived sensitivity); the type of user requesting their data to be shared, including their perceived motivation, profit prioritization, and ability to directly impact patient care; trust in the data user, as well as in associated processes, often established through individual choice and control over what data are shared with whom, when, and for how long, supported by appropriate models of dynamic consent; the presence of a feedback loop; and clearly articulated benefits or issue relevance including valued incentivization and compensation at both an individual and collective or societal level.

**Conclusions:**

There is general, yet conditional public support for sharing personal health data for third-party or secondary use. Clarity, transparency, and individual control over who has access to what data, when, and for how long are widely regarded as essential prerequisites for public data sharing support. Individual levels of control and choice need to operate within the auspices of assured data privacy and security processes, underpinned by dynamic and responsive models of consent that prioritize individual or collective benefits over and above commercial gain. Failure to understand, design, and refine data sharing approaches in response to changeable patient preferences will only jeopardize the tangible benefits of data sharing practices being fully realized.

## Introduction

International advances in information communication, eHealth, and other digital health technologies have led to significant expansions in the collection and analysis of personal health data [1]. While the benefits of data sharing are widely recognized, including improved care quality [2-4], strengthened care coordination [3], reduced medical costs [4], and enhanced treatment development [5], critical exploration of public attitudes, perceptions, and preferences remains limited [1,3,4,6,7]. Furthermore, as reported by Braunack-Mayer et al [8], a specific focus on public preferences for sharing personal health data with third parties or for secondary uses (ie, other than their health care) remains sparse [9].

Critical exploration of such perceptions is considered vital as the optimization and successful execution of data sharing relies on public support [4,10]. Furthermore, to advance existing knowledge and understanding, health care systems are increasingly partnering with third-party organizations to provide novel data processing and analysis capabilities [2]. Moreover, several high-profile data sharing scandals, including the Cambridge Analytica scandal [11], period tracking app scandals [12], and other scandals related to the National Health System [13] have arguably heightened public concern.

Developing a sound understanding of public willingness to share personal health data with third-party organizations or for secondary uses is therefore essential if we are to leverage the full potential of data sharing practices and inform international policy [14]. In addition, following the COVID-19 pandemic and possible related changes in public willingness to share health data, an updated synthesis of available literature is urgently required [15].

Thus, while existing research has often focused on data sharing in a single area, such as research [9,16], health administrative or clinical trial data [17], or electronic health records (EHRs) [7,18], this review aims to synthesize previously siloed literature into a single corpus of information, identifying barriers and enablers that affect public willingness to share personal health data for third party or secondary uses.

## Methods

### Overview

A systematic review following the PRISMA (Preferred Reporting Items for Systematic Reviews and Meta-Analysis; Multimedia Appendix 1 [19]) guidelines [20] and guidance by Popay et al [21] on the conduct of narrative synthesis in systematic reviews was conducted. This systematic review sought to address the following review question: What factors affect public willingness to share personal health data for third-party or secondary uses? A review protocol was not registered, but the review was assessed by an information specialist according to PRESS (Peer Review of Electronic Search Strategies) guidance [22].

### Search Strategy

Following the PRESS guidance [22], the search strategy was informed and approved by an information specialist. Six databases were searched: MEDLINE, Embase, PsycINFO, CINAHL, Scopus, and SocINDEX. Search terms used (Table 1) were designed to maximize sensitivity and specificity using the SPICE (Setting, Population [or perspective], Intervention, Comparator, and Evaluation) framework [23]. Database searches (conducted August 11, 2021) were also supplemented with reference list searches to ensure sufficient coverage.

**Table 1:** Search terms used organized according to the SPICE (Setting, Population [or perspective], Intervention, Comparator, and Evaluation) framework.

	Search terms
Setting	“health data” OR “personal health data” OR “health information exchange*” OR “wearable* data” OR “patient data” OR “personal data” OR “personal health information” OR “patient health record*” OR “patient record*” “electronic health record*” OR “personal health record*”
Perspective	consumer* OR patient* OR client* OR citizen* OR carer* OR user* OR “end user” OR public* OR stakeholder* OR service-user* OR “service user*”
Intervention	[share or sharing or transfer* or access* or “secondary us*” or link] adj3 [data or record* or information]
Comparison	N/A^a^
Evaluation	attitude* OR perspective* OR willing* OR perception* OR barrier* OR enable* OR facilitat* OR opinion* OR trust* OR confiden* OR concern* OR view* OR challenge*OR accept* OR qualitative OR interview* OR survey*

^a^N/A: not applicable.

### Study Selection

Studies were selected through a 2-stage process. First, due to the large number of abstracts returned, studies were equally split among 5 reviewers (RB, SS, DA, KA, and HB; n=201, 20% each) who independently reviewed returned abstracts for study inclusion using a collaboratively agreed decision-making flowchart and predefined inclusion and exclusion criteria outlined below. To ensure consistency and decision-making rigor, 10% of each reviewer’s abstracts were randomly selected to be blindly assessed and compared by a second reviewer. This process was facilitated using Rayyan (Rayyan Systems Inc), a web-based app for systematic reviews. If an inclusion decision could not be made from the abstract alone, the paper was taken through to full text. Papers included in this first stage were then read in full and independently assessed for study inclusion by the research team. Reasons for study exclusion were recorded during this process for transparency purposes. If discrepancies in inclusion or exclusion decisions could not be resolved by discussion alone, this would have been resolved by being sent to a third reviewer until consensus was achieved. However, this process was not required in this review.

### Inclusion Criteria

Papers published in the English language between 2011 and 2021 that explored patient and public attitudes toward sharing personal health data with third-party organizations or for secondary use, using any study design except protocols, conference proceedings, letters, or theses were included. Justification for the data parameters used stems from the rapidly evolving nature of technology acceptance, data sharing policies [24], and the desire to ensure only the most contemporary information was included. For clarity, if a paper discussed views from patients and the public and other related stakeholders such as researchers, the paper was included if the source from which the data were being presented was clear.

### Exclusion Criteria

Protocols, conference proceedings, letters, or theses, not available in the English language, published prior to 2011 that did not explore patient and public attitudes toward sharing personal health data were excluded. Studies that discussed patient and public attitudes toward sharing personal health data by proxy, for example, from the perspective of health care professionals alone were also excluded due to the study’s focus on understanding public attitudes toward sharing personal health data and not related assumptions or perspectives. If it was not possible to identify the source from which the data were being presented, that is, patient or health care professional, the paper was excluded. Due to limited resources, a sensitive interpretation of non-English papers could not be guaranteed. Non-English papers were therefore excluded, even though this may have introduced a risk of bias.

### Data Extraction

To ensure consistency and standardization, 6 reviewers independently undertook data screening and extraction using a piloted data extraction form. The information extracted from the papers encompassed details such as author names, publication dates, study locations, study designs, data types, recipients of the data (whether individuals or organizations), as well as factors influencing the sharing of personal health data. For clarity, only information that was clearly identified as being from a patient or public perspective was extracted. No risk of bias assessments were undertaken due to the size of the literature reviewed.

### Data Analysis and Synthesis

Review findings were analyzed using inductive thematic analysis as proposed by Braun and Clarke [25]. Defined as a method for identifying, analyzing, and interpreting patterns of meaning (themes), inductive thematic analysis embodies an organic approach to coding and theme development [25,26]. Justification for adopting an inductive thematic approach includes its provision of an accessible and systematic procedure to generating codes and themes [26], emphasis on producing rigorous analyses, and providing flexibility in identifying patterns within and across multiple data types [26].

Identified themes were synthesized using a narrative approach defined as “an approach to the systematic review and synthesis of findings from multiple studies that relies primarily on the use of words and text to summarize and explain findings of the synthesis” according to the guidance mentioned in Popay et al [21]. Other systematic reviews have adopted a similar approach to data analysis and synthesis [27,28], justifying its inclusion in this review. Simple tables of themes have also been created to summarize findings wherever possible. No sensitivity or certainty assessments were conducted.

## Results

### Overview

**Figure 1:**
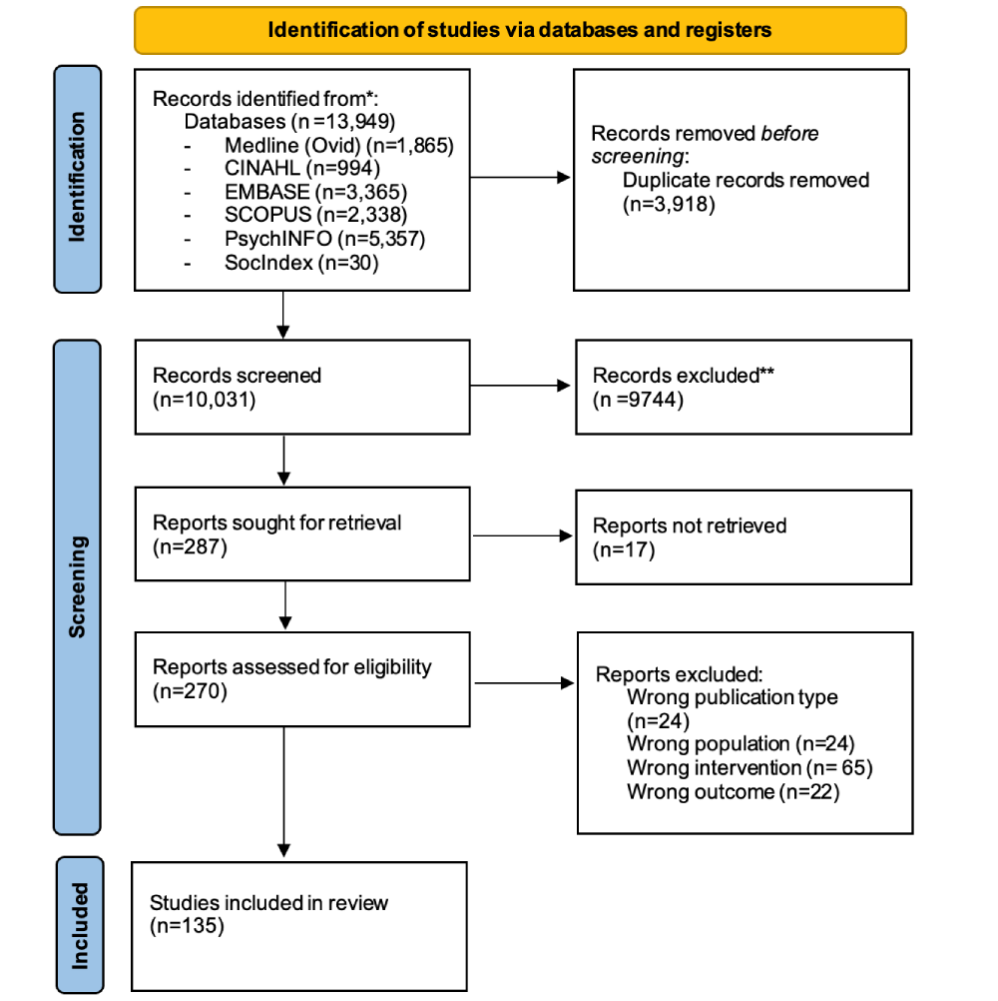
PRISMA (Preferred Reporting Items for Systematic Reviews and Meta-Analysis).

Of the 13,949 papers originally identified, 135 were included (Figure 1). Included papers discussed the opinions of 164,478 patients or public members and reviewed 173 papers. Most papers were published in the past 5 years from the United States (n=64), the United Kingdom (n=18), and Canada (n=9), with representation from 17 other countries and continents including Ghana, Taiwan, Japan, Egypt, and Australia.

As shown in Multimedia Appendix 2 [2-4,6,7,9,10,12,14-18,24,29-147] and Multimedia Appendix 3, most papers were of a survey (n=68) or qualitative (n=41) design, with mixed methods (n=12), reviews (n=6), citizen juries (n=5), discrete choice experiments (n=1), randomized controlled trial (n=1), and experimental (n=1) study designs also used. Most papers discussed a willingness to share personal health data (n=52), EHRs (n=31), or genomic or genetic data (n=12), with either researchers (n=61), health care providers (n=27), or multiple stakeholders combined (n=38). Other types of data reviewed included app data (n=2), monitoring data (n=2), adverse drug events (n=2), and personal health data voluntarily shared digitally (n=1).

The majority of papers explored willingness to share data from a patient (n=47), patient and public (n=48), or public (n=26) perspective, with patients and caregivers (n=5), parents (n=1), next of kin (n=1), employees (n=1), and insurance customers (n=1) also represented (Multimedia Appendix 3). For clarity, only data pertaining to patients (inclusive of parents and caregivers) and public were extracted. Of the included papers reviewed, 2 had a focus on rare diseases. A table of study characteristics is provided in Multimedia Appendix 2 [2-4,6,7,9,10,12,14-18,24,29-147] with further summary tables also available in Multimedia Appendix 3.

Reflecting the review’s aim, the results are synthesized according to key themes that both support (enablers) and inhibit (barriers) public willingness to share personal health data for third-party or secondary uses.

### Barriers

#### Data Protection

Beginning with barriers, perhaps unsurprisingly, data privacy, security, and management concerns were the barriers most commonly identified as affecting public willingness to share personal health data [3,4,9,14,16-18,29-55,148]. Such findings are concerning, as several studies highlighted the detrimental outcome of patients intentionally engaging in privacy-protective behaviors including withholding clinically relevant information from health care providers to counteract data privacy and security concerns [48,56,57]. For example, one study stated the following:

If individual needs for privacy and security of data exchange are not met, consumers may intentionally prefer to hide relevant health information from their healthcare providers.

56

In some studies, data privacy was the most influential factor affecting willingness to share [4,51,52,58], highlighting the severity of the issue at hand.

Specific data privacy and security concerns identified included confidentiality breaches [16,59] often linked to the risk of reidentification [16,60], or ineffective anonymity processes [55,61]; unauthorized, or unknown data access [18,39,42,44-46,51,62,63]; data misuse and abuse [3,15,16,34,47,55,59,63,64], particularly for stigmatizing, or sensitive health conditions [45,47,55]; data or identity theft and fraud [7,15,16,18,31,39,44,58,63-67]; and the unauthorized reuse or future use of collected data [15,16,18,42,59,60,65,68] that extends beyond the scope of originally intended and consented purposes [59,68]. The latter point indicates how data sharing practices often operate in “largely unchartered territory and, as such, new harms may emerge that we cannot yet foresee” [65], accentuating the importance of dynamic consent that enables progressive patient choice as later discussed [47].

Concerns that data sharing activities may adversely affect an individual or community [2,15,49,59,60,67] also appear to have a significant impact on public willingness to share personal health data. Several papers discussed the risk of exploitation [3,16,44,60,65], inequalities [3,44]; “fear of discrimination” [18] or stigmatization [3,15,16,18,30,44,47,60,65-67,69-71]; financial hardship including insurance premiums being increased or denied [18,59,64-67,71-73]; reduction in care quality [31] or person-centered care [3]; employers having oversight of personal information [58,59,64,74]; and feelings of judgment, shame, or guilt [14,75]. Concerns of adverse consequences appeared particularly salient when being asked to share more sensitive or stigmatizing data [24,47,59]. However, as later described, what constitutes sensitive data is largely dependent on individual perceptions, definitions, and experience.

Concerningly, confidence in existing data security systems and processes appears low [44,58,64,76,77]. For example, only 38.4% (n=19,372) of respondents agreed that health care providers are currently successful in providing effective data security [77]. The majority of US citizens do not trust organizations that store and share their personal health data [76]. The size and bureaucracy of an organization have also been linked to a perceived inability to protect personal health data [36,44], with 71.3% (n=1969) of participants stating that they could not fully trust the “big and bureaucratic” National Health System to guarantee the security of EHRs [44]. Size and status do not therefore appear to guarantee public trust and confidence. Alternative ways of nurturing public trust that align with public preferences and expectations may therefore be required, as health care systems and organizations enter a rapidly evolving era of expanded data sharing [76].

#### Public Awareness and Understanding

Other factors that undermine public willingness to share personal health data include limited public awareness and understanding [16,18,29,51,53,59,60,71,78-84]. Studies often reported that most patients feel they are not receiving the information required to understand data sharing opportunities [85] and thus provide informed consent. Participants also seemed to be unaware of the meaning and value of their health data to third-party organizations [86]. However, O’Brien et al [58] found that of the 693 patients who were not comfortable with sharing deidentified data, most reported that their comfort levels would increase if they were made aware and understood how their health data were protected [58]. Such findings reiterate the combined effect of increased public awareness and understanding and assurances of data privacy and security in facilitating public support.

#### Data Type

In addition to awareness and understanding, willingness to share personal health data also appears dependent on the type of data being shared [18,24,34,61,70], in particular, whether data are considered “sensitive” or not. While sensitive data are often clearly defined in privacy regulations such as the General Data Protection Regulation, participant definitions of sensitive data often differed between and within studies. Types of data identified by participants as particularly sensitive or stigmatizing are listed in Table 2.

**Table 2:** Identified types of sensitive or stigmatizing data.

Data type	Supporting references
Mental health	[18,32,33,43,59,64,67,77,87]
Sexual health including reproductive health	[18,32,33,43,59,77,88]
Genetic or genomic data	[15,16,33,66,67]
Substance use	[18,33,77,87]
Social security number and insurance ID	[51,58,89]
Financial information	[32,34,51]
Sexuality	[59]
Religion	[59]
Disability status	[64,66]

However, while similar categorizations of sensitive data types exist, diversity in patient-defined classifications and data sharing preferences persist [87], particularly regarding genetic and genomic data [18,70,90]. For example, research conducted on rare disease perspectives concludes that roughly half of individuals consider information on their disability (51/47), genetics (49/48), or physiology (48/50) to be sensitive. However, this also means that half of the respondents did not consider such information to be sensitive [66]. Furthermore, in a study exploring data sharing among children with genetic conditions, parents of typically developing children reported being uncomfortable sharing sensitive information and in some cases, completely unwilling to do so. Conversely, parents of children with Fragile X syndrome and autism spectrum disorder were willing to share their child’s sensitive data including mental health and genetic information [67]. Similar variations in data sharing preferences depending on rare disease or health status results have also been reported in cancer, where patients with cancer reportedly more willing to share sensitive genetic data [91]. While the direction of this effect was opposite to that originally hypothesized, it was the inherited genetic information that often made participants with cancer more willing to share such data [91]. Thus, emerging evidence suggests individuals with inherited or rare diseases may be more inclined to share personal health data to advance existing knowledge and treatment options, with the term “informational altruists” often used [90]. A universal definition of sensitive and nonsensitive data is therefore not available given variations in peoples’ perspectives, health status, and experience.

#### Type of Data User

##### Overview

In addition to data type, the type of user requesting data to be shared also appears highly influential in determining public willingness to share personal health data [82,92,148,149]. Most patients appear more willing to share health data with health care providers or organizations [24,32,92-95], researchers [32,67,70,96-98,149], including researchers at universities [14,18,32,40,55,67,70,92,99-101], although not always [59,77], researchers who identify as medical professionals [10,59,92,95] and nonprofit organizations [18,40,67,70], as they trust such entities to “produce socially valuable knowledge, products and interventions” [55] that are often meaningfully constrained by ethics and ethics boards [67].

Conversely, patients appear less willing to share personal health data with government bodies [18,59,102], private insurance companies [16,32,34,36,44,54,67,72,77,84,92,95,100] (often linked to a fear of being denied coverage [67,72,101]), pharmaceutical companies [14,16,18,40,67,68,77,84,92,96,103], and commercial or private entities [16,32,36,40,44,47,51,59,60,67,69,70,97,100,102,104-108]. However, it is often not a straightforward delineation between data user type and public willingness to share. Such relationships are often complex, dynamic, and highly variable [17,34,41,55,71,76,82,96,109,110], depending on personal attributes or personality traits, the purpose, context, perceived benefit, incentivization, and compensation of data sharing requests. As Aitken et al [59] mentioned, “there does not appear to be a clear, or static hierarchy of trusted organisations/sectors.” Understanding and responding to public preferences for who, when, how, and under what circumstances they share their personal health data are therefore imperative.

##### Perceived Motivation of Data User

A common factor underpinning public willingness to share personal health data is the perceived motivation of the data user type [14,61,96], with the end purpose, or intended use considered to be highly influential in determining willingness to share personal health data with a third party [14,96]. This is perhaps most evident in data sharing with pharmaceutical, insurance, or commercial companies [93,96]. Specifically, the perceived prioritization of profit [14,16,54,59] over individual or collective health and well-being [59]. For example, recent research conducted by Jagsi et al [111] reported that when insurance companies were the data receiver, 79.5% (n=171) of participants felt comfortable if the purpose was to ensure patients received recommended care. However, this figure dropped to 50.9% (n=110) if the data were being used to determine insurance eligibility or reimbursement [111]. Similarly, when drug companies were the intended data users, most participants were comfortable if the information was being used to help develop new treatments (n=200, 92.6%) or understand which patients may benefit from certain drugs (n=197, 92.1%) [111].

Included papers suggest patients are increasingly recognizing the balance of working with pharmaceutical and private companies [59]. Mamo et al [54] mentioned that participants were supportive of “corporate profits if the company developed new or modified medical technologies that would help people and if such help could be distributed equitably.” Profit creation does not therefore necessarily dictate the automatic refusal of private sector involvement [2,59,107,112]. Profit creation can be seen as acceptable under certain conditions [2,107,112]. Namely, when the intended purpose is of clear public benefit [107,112] (a notion referred to as the “public benefit criterion” [107]), there is a transparent commitment to sharing or appropriately reinvesting profits to create further public or societal benefits [59,112], and any commercial gain that is accrued is secondary to ensuring public benefit [107], that is, public benefits are prioritized above profit [112]. Thus, social responsibility, evidence of equitable and reciprocal benefits, and the prioritization of public benefit over commercial gain are widely regarded as an essential prerequisite for public support [59].

##### Distance Between Provider and Data User

Finally, linked to perceived motivation is the perceived distance between the data requester and their ability to directly impact patient care. The further data recipients appear from having a direct impact on care, the less willing patients are to share their personal health data, often under the inference that there is no clear reason why individuals need access to such data [95]. Clearly articulating the purpose behind data sharing exercises, particularly when there is a significant “distance” between data requester and patient impact, also seems imperative.

### Facilitators

#### Personal Attributes or Personality Traits

With regard to facilitators, several personal attributes or personality traits appear to support a willingness to share personal health data. Those most frequently identified are shown in Table 3.

**Table 3:** Personal attributes and personality traits that support a willingness to share personal health data.

Personal attributes and personality traits	Supporting references
Age: Although opinion appears divided on the direction of influence, with some researchers suggesting older individuals are less willing to share, others suggest they are more willing to share, with some authors reporting no difference in age and willingness to share	[2,15,16,32,33,40,44,58,60-62,66,70,76,97,99,100,102,113-117,149,150]
Educational attainment: Evidence to suggest people with a lower level of educational attainment are typically less willing to share health data, although this conclusion is again not unanimous, with evidence to suggest the impact of educational attainment may also be dependent on the purpose of any data sharing activities	[2,15,16,32,33,44,48,58,64,66,70,100,101,109,114,118-120,150]
Sex: Differing conclusions on whether men are less or more concerned than women about data sharing	[16,32,60-62,64,66,88,100,102,113,114,150]
Ethnicity: Black participants are often less willing to share personal health data than White participants	[16,44,76,90,96,100,101,109,114,118,119]
Health status: Severity and rare disease, with healthier individuals typically more willing to share personal health data	[15,40,58,62,66,113]
Issue involvement or perceived level of personal relevance and benefit: Higher issue involvement or personal relevance typically yields greater support	[4,6,7,31,39,44,49,51,52,67,69,105,115,120-123]
Privacy concerns including data misuse and fear of harm: Fewer privacy concerns associated with greater intentions to share	[2,6,40,44,52,62,63,113,115,120,122]
Religious beliefs: Lower religiosity is often associated with a greater willingness to share	[15,16,102,109]
Employment status: Being employed is often associated with a greater willingness to share	[33,61,64,76,115,150]
Household income	[35,63,115,120]
Internet or computer access and smartphone ownership or familiarity (sometimes referred to as digital health literacy): Individuals more comfortable using digital technologies are typically more willing to share personal health data	[7,35,52,62,63,100,115,124]

Other factors identified as influential included the presence of altruistic traits [49,58,120]; generalized trust [49,120] or high levels of trust in health care providers [6,7,120,122]; health service use, with more frequent users more likely to be supportive of data sharing [49,114]; patient activation [4]; patient-physician relationship [4]; nationality [15,33]; health insurance coverage [120], with individuals experiencing cost barriers to care typically less willing to share personal health data [96]; marital status [15,16,63]; parental status [15]; child health status [63], with parents of children in excellent-to-good health status less likely to share health data [63]; existing knowledge or personal experience of data sharing topic [15,100], particularly in relation to genetics or genomics [10,71,102] (although not always [70]); and previous experience of research participation [58,89].

Often several personal attributes affected data sharing willingness simultaneously. For example, evidence suggests that Black, Asian, and minority ethnic populations [90,100,114], younger age groups [100], and those with lower education attainment [100] are generally less supportive of data sharing. However, it is important to note this is not always the case [44,66], with other factors identified including data user type and purpose also influencing intentions to share, highlighting the complexity of data sharing intentions and the subsequent importance of understanding and aligning data sharing requests with public expectations and requirements.

#### Clear Demonstration of Data Sharing Benefits

Another key factor in supporting data sharing willingness is the clear demonstration of personal, public, or knowledge development benefits. Table 4 outlines the potential benefits of sharing health data ordered according to the frequency in which they were reported in the included literature.

**Table 4:** Potential benefits of sharing personal health data ordered according to reporting frequency.

Reported benefit of data sharing	Supporting references
Personal health benefits include helping health care professionals make better decisions about their health through increased awareness and understanding	[4,9,14,16,18,29,39,44,46,52,54,58,59,67,83,106,115,122]
Contributing to the “greater good” or altruistic sharing for public benefit	[9,14,16,29,32,48,55,59,63,67,74,105,106,122,125]
Improved care quality, including enhanced treatment options or quality, personalized care provision, improved diagnosis accuracy, and speed	[16,18,41,42,48,52,54,77,115]
Facilitating innovation development, particularly making new therapies available quicker	[16,58]
Reducing resource or research wastage	[16,44]
Increased access to information including sharing of research findings	[41,105]
Reducing treatment delays and medical errors	[41,77]
Preventing health epidemics	[16]
Enabling the study of rare events	[16]
Improving research quality	[32]

Some research provides additional insights into which benefits are considered most desirable from a patient perspective. For example, research conducted by O’Brien et al [58] suggests that the highest proportion (n=3305, 94%) of patients stated that “Helping my doctor make better decisions about my health” and “Helping make new therapies available faster” were “extremely” or “very important” benefits associated with data sharing. “Helping researchers evaluate the quality of care delivered by doctors and hospitals,” “Helping other patients with my primary health condition,” and “Reducing the cost of doing research” were also rated as “extremely” or “very important” by the majority of patients (91% and 89%, respectively) [58]. Furthermore, patients reported the strongest level of statement agreement with “knowing the study using my data could help patients with my health condition” (mean summary score 1.44) and “knowing the study using my data could reduce health disparities” (mean summary score 1.54) [58]. Patients were less likely to agree that comfort levels would be improved by “knowing the study using my data could help patients with other health conditions” [58]. Similar results have been reported elsewhere [16,18], reiterating the importance of issue involvement or personal relevance previously discussed, suggesting data sharing intentions may increase if there is a direct association between data sharing requests and assured advances in knowledge, treatment quality, and options for the health condition individuals experience. However, it is also important to note that many individuals also appear motivated by the concept of contributing to the “greater good” or altruistic sharing for public benefit [9,14,16,29,32,48,55,59,63,67, 74,105,106,122,125]. Therefore, highlighting the purported benefits of sharing health data on both individual and collective levels may be beneficial.

#### Trust (Specifically, How to Build It)

##### Overview

Underpinning many of the barriers and enablers listed above is trust, something that can be earned and irreparably damaged [112]. Many papers described the importance of trust in facilitating the sharing of health data [20,45,47,48,51,54,55,84,95,112,120,123,126,149]. However, few studies specifically described how to establish it. The following subthemes therefore report on available literature that supports the development and maintenance of trust.

##### Control

A core feature of trust in data sharing processes is control [16,18,32,55,66,73,74,79,84,86,92,104,127,128]. Specifically control over what data are shared, with whom, and when, with the majority of participants “overwhelmingly in favour of keeping the strictest control on their data” [66], believing “they have a right to control the use of their data” [127]. For example, in 1 study, all participants (100%) stated that they would like to be able to control what entities access their EHR [79]. Perceived control also appears to lower privacy concerns [59,62,69,104], with clear public preferences for controlling the type, duration (eg, not for the duration, or any longer than the research study [43]), and level of access depending on different data user types or individuals or organizations and data sharing purposes [18,44,73]. Thus, as noted by Courbier et al [66], being in favor of data sharing practices and wanting more control are not contradictory but rather parallel requirements.

As alluded to below, granular or hierarchical control [79,86,104] is likely to “become more and more important in the practice of healthcare technology and services” [88]. However, it is also important to acknowledge variations in desired levels of control [79]. Adopting a personalized approach to data sharing control is therefore required. Without patients feeling in control of their data sharing practices, the likelihood of nurturing and sustaining data sharing intentions is minimal.

##### Choice

In addition to control, individuals also require choice in how, what, and when their data are both accessed and shared [16,24,70,79,88,104]. Both elements (choice and control) are considered “crucial for the public to be able to decide who and how to trust” [129]. However, many participants do not yet feel that they have control and choice over their own data, including sensitive data such as biomedical data [129]. Caine et al [79] provided six implications for the design of a patient-centered tool that enables individual choice in the disclosure of personal health data: (1) easy patient access, (2) an overview of current sharing permission, (3) granular, hierarchical control over data access, (4) access controls based on dates, (5) contextual privacy controls, and (6) notification of when their data have been accessed [79].

##### Consent

Further strengthening the relationship between trust and choice is consent [18,127], with consent often acting as a mechanism for facilitating individual control [59]. While consensus on public preferences for models of consent (eg, explicit opt-in, opt-out (often least favorable), broad, 1-time consent, project specific consent) is not yet available [54,56,119], varied, dynamic, or flexible consent [47,59,66,67] that enable individuals to change their consent preferences over time appears most desirable, recognizing public preferences can often change in response to events, project purposes, motivations, and incentivization [59,78]. Dynamic models of consent may also provide beneficial flexibility and adaptability in response to future technological and regulatory, legal changes [66]. Appropriate models of consent appear particularly important in relation to identifiable data, qualitative information, genetic research, or when a commercial entity is involved [59,98], mirroring previous barriers identified above.

Modernizing consent policies to better reflect changing public preferences is therefore also considered crucial in facilitating transparency and fostering sustained patient engagement and empowerment [90,97].

##### Feedback Loop

Other mechanisms for establishing trust and transparency include the provision of a feedback loop [47,67]. Such practice was often seen as a form of “compensation” or expression of appreciation and recognition [51]. However, the creation of a feedback loop can be onerous and is often dependent on the use of identifiable data. Despite this, relaying the impact of data sharing is considered “a matter of respect” [67] and a highly desirable way [15,88,93,97,104] to “improve trust and public engagement” [47]. In 1 study, 99.7% of participants positively responded that they would like to be informed about the outcome of a data sharing project they participated in [15], with the ideal frequency of being informed for the majority of respondents being once a month [66]. Developing a clear feedback loop was therefore repeatedly identified as a way to encourage and support data sharing practices [71].

##### Assurances of Patient Confidentiality and Data Protection

Other ways to develop trust and subsequent data sharing support include providing assurances of data privacy, security, and confidentiality [10,17,55,59,64,74,83,126,130], with assurances of confidentiality often associated with aggregated, or trust in the data’s anonymization processes [16,33,47,58,59,61,63,64,113,148]. The influence of governance and safeguarding assurances on public acceptance should not be underestimated [20,43,44,59,116]. Existing literature suggests that it is insufficient to simply indicate that participant data will be kept securely. Specific information regarding what data are being collected, where the data will be stored, which safeguards are applied, who has access to the information, and how data breaches or misuses will be addressed [55,67,71,99,103] is required. Informing the public about security practices and processes can help reassure patients that data sharing is being responsibly executed in a framework of accountability [54,67,112]. More transparency and detailed information regarding data usage and protection are therefore urgently required, ensuring such information is included in consenting content.

#### Transparency

##### Overview

In addition, providing transparent information about who will benefit from data access has also been identified as the most important measure to increase trust, having been endorsed by more than 50% of participants across 20 of the 22 countries studied, followed by transparency about who is using data and why [123].

##### Incentives and Compensation

Finally, the provision of meaningful incentives and compensation [10,51,72,86,93,105,121,148,149] “broadly defined to include financial compensation, expressions of appreciation...recognition and sharing of results” [51] also appears integral in fostering public support. Recent research conducted by Luo et al [14] identified the following types of compensation: free treatment, money, food, cryptocurrencies, discounts on health insurance, shared research findings, and donations to a good cause, with free treatment receiving the most mentions, followed by cryptocurrencies and money. Furthermore, as noted by Navarro‐Millán et al [124], self-tracking technologies and data sharing requests may be more appealing if “coupled with opportunities to learn” about specific health issues including symptom management, medications, side effects, and opportunities to obtain social support. Perhaps unsurprisingly, the majority of participants report that they would be “more” or “much more” likely to share data if they felt appropriately compensated for their actions [105]. For some, individuals are also incentivized by the idea of benefiting themselves or their community [121], reflecting altruistic motivations [47,51,59,67,74,82,149] previously described. However, appropriate compensation (recognizing this looks different for different people) appeared particularly influential if the third party, particularly pharmaceutical companies were making money from participant data [14], with resulting profits expected to be returned to the public or patient [108]. Providing valued, reciprocal, and equitable benefits therefore appears influential in facilitating data sharing intentions [72].

## Discussion

### Summary of Findings

This review sought to explore factors that affect public willingness to share personal health data for third-party or secondary uses. Perhaps unsurprisingly, data privacy, security, and management concerns were most commonly identified as the biggest barriers to sharing personal health data from a public perspective. Other factors found to influence public willingness include the type of data being collected (ie, data sensitivity); the type of data user requesting data to be shared, including their perceived motivation, profit prioritization, and ability to directly impact patient care; personal attributes or personality traits; trust established through individual choice and control over what data is shared with who, when, and for how long, supported by appropriate models of dynamic consent; the presence of a feedback loop, and evidence of perceived benefits or issue relevance including valued incentivization or compensation at both an individual and collective or societal level.

Profiting from data sharing does not necessarily dictate public rejection. Namely, when the intended purpose of sharing data is of clear public benefit, there is a transparent commitment to sharing data or appropriate reinvestment of profits to create further public or societal benefits, and any commercial gain is secondary to ensuring public benefit, concerns appear reduced but not entirely removed. Thus, social responsibility, evidence of equitable and reciprocal benefits, and the prioritization of public benefit over commercial gain are widely regarded as an essential prerequisite for public support.

Conversely, universal agreement on patient definitions of sensitive data is not yet available, with variation in patient data sharing preferences also apparent including the perceived direction of influence regarding certain personality traits or personal attributes. Such findings reiterate the complex context data sharing practices often operate within and the subsequent need for more dynamic and responsive models of consent that provide individual choice and control over who has access to what data, when, and for how long. Without patients feeling in control of their data sharing practices, the likelihood of nurturing and sustaining data sharing intentions is minimal.

### Comparison With Existing Research

Many of the factors identified as influential in this review including data privacy and security, data, and user type have been reported in previous literature [16,131,151]. Thus, previous conclusions of widespread, yet conditional public support for sharing health data appear to remain true [16]. There was also no reported difference in themes identified in pre- and post–COVID-19 publications. Further exploration of such comparisons may be beneficial moving forward as post–COVID-19 literature continues to emerge and develop. However, this review provides previously unavailable insights into the factors that influence public willingness to share personal health data across historically siloed literature, with several factors (eg, privacy, value, and transparency) clearly synonymous across different patient data sharing fields.

Novel contributions of this review include its synthesis of updated literature following COVID-19 and conscious focus on trust, specifically how to build and nurture trust against a highly changeable background of public preferences and expectations. Focusing on more modern models of dynamic consent that engender individual choice and control, this review provides additional insights into the building blocks of establishing trust in the complex context of sharing personal health data for third-party or secondary uses.

### Implications

The implications of this review are clear. First, there is a need to reconceptualize the public as active data owners and not as passive data subjects or providers [59,74]. Second, regular attention needs to be paid to the changeable conditions of public support, consciously designing and refining data sharing processes and related policies to reflect ongoing public preferences and requirements [48,70,86,91,98,122]. While the immediate cost of mishandling personal health data can be calculated following high-profile scandals [152,153], the longer-term impacts of failing to respond and support public willingness to share personal health data are not yet known. Third, every effort should be made to ensure the purpose and details of data sharing requests including assurances of data privacy and security, data and user type, personal and collective benefit, profit, and duration (ie, no longer than required for the originally consented activity [43]) of data sharing processes are clearly articulated [154], with individual members able to continuously choose and refine desired levels of control through accessible consenting processes. Part of this involves actively raising awareness of data sharing processes in a bid to build public trust and support [7,44,59]. However, it is imperative that awareness raising is not approached as a simple 1-way exchange of information. A more engaged approach that supports opportunities for ongoing deliberation regarding public interests, concerns, and uncertainties, as opposed to controlled dissemination, is required [59]. Providing public information does not equate to enhanced public trust [106,107,125]. As suggested by Middleton et al [71], suggested areas of information include the purpose of data sharing, why partnerships between profit and not-for-profit industries are necessary, and what the relevance of data sharing processes is to public lives. But first, we must work to understand what people want to know and how to make the subject resonate [71], ensuring opportunities afforded by data sharing practices are not miss-sold or overemphasized in a bid to engender public support, highlighting the ethical arguments that underpin such activities. Similar to seeking patient feedback about experiences of care [155], it is perhaps unethical to ask patients to share their personal health data if no change or progress is to be achieved. Failure to use consented data sharing practices only seeks to invalidate and undermine patients’ time and expertise. Understanding public needs and aspirations around these issues is arguably best achieved through meaningful involvement or co-design to ensure information value, relevance, and understanding [3,54]. As stated by Franklin et al [82],

“It is vital that we understand patient perspectives on data sharing and work with them as partners, valuing their unique contributions and attending to their preferences.”

Working with patient advocacy groups may be an essential component of this process, particularly in areas where public confidence in patient-sharing initiatives may already be weakened.

Finally, public trust is widely recognized as the central pillar in supporting public willingness. However, few studies have explicitly explored how to build public trust. Emerging research suggests health care organizations and institutions may be able to increase public comfort by sharing health data with third-party or commercial companies by emphasizing patient-centered benefits [2]. However, future research including the application of trust models may further benefit from focusing on how issues and relationships of trust can be both nurtured and overcome [59].

### Strengths and Limitations

Strengths of this research include its application of a recognized systematic review process, the inclusion of updated literature following COVID-19 including international literature from developing countries, responding to criticisms that existing studies have largely concentrated on developed countries only [62], and synthesis of historically disparate literature into 1 corpus of information. However, its limitations must also be acknowledged. Much of the existing literature is based on data sharing intentions, or hypothetical scenarios as opposed to real-world, or observed behavior [4,32,52,57,73,96,101,109,113]. Reported findings may not therefore translate into practice [10,71,132]. However, commonality in findings provides some level of confidence in the findings presented. Furthermore, there is evidence to suggest people are more hesitant about hypothetical scenarios than they are in reality [10,71,102,156]. Moreover, not all included studies focused on intentional behavior, with some exploring data sharing practices in real-world settings [18,118]. Finally, the literature search was restricted to the English language only. The risk of publication bias is therefore acknowledged. Further research may also benefit from a critical exploration of country-specific legal restrictions and their role in data sharing practices.

### Summary

In summary, there is general, yet conditional public support for sharing personal health data for third-party or secondary use. Clarity, transparency, and individual control over who has access to what data, why, when, and for how long are widely regarded as an essential prerequisite for public data sharing support. Individual levels of control and choice need to operate within the auspices of assured data privacy and security processes, underpinned by dynamic and responsive models of consent that prioritize individual and collective benefits over and above commercial gain. Failure to acknowledge, design, and refine data sharing processes in response to patient preferences and needs will jeopardize the tangible benefits of data sharing practices being fully realized.

## Appendix

Multimedia Appendix 1 PRISMA (Preferred Reporting Items for Systematic Reviews and Meta-Analysis) 2020 checklist.

Multimedia Appendix 2 Table of study characteristics.

Multimedia Appendix 3 Summary tables.

## Abbreviations

EHR: electronic health record
PRESS: Peer Review of Electronic Search Strategies
PRISMA: Preferred Reporting Items for Systematic Reviews and Meta-Analysis
SPICE: Setting, Population (or Perspective), Intervention, Comparator, and Evaluation

## Data Availability

The data sets generated during and analyzed during this study are available from the corresponding author on reasonable request.
