# Supplementary material for: Patient and Public Willingness to Share Personal Health Data for Third-Party or Secondary Uses: Systematic Review
Source: J Med Internet Res. 2024 Mar 5;26:e50421. doi: 10.2196/50421 (PMC10951832; doi:10.2196/50421)
Supplement: Multimedia Appendix 2 [file jmir_v26i1e50421_app2.docx]

| **Author name** | **Year** | **Perspective** | **Sample size** | **Specific population** | **Setting** | **Study design** | **Type of data** | **Purpose/intended sharer** |
| --- | --- | --- | --- | --- | --- | --- | --- | --- |
| Abdelhamid et al., | 2017 | Patients and/or public | 1606 |  | US | Survey | Personal health data | Health care providers |
| Adu et al., | 2021 | Patients and/or public | 276 |  | Ghana | Survey | Personal health data | Health care providers |
| Aggarwal et al., | 2021 | Patients | 408 |  | UK | Survey | Personal health data | Research |
| Aitken et al., | 2016 | Patients and/or public | 25 papers |  | International | Systematic review | Personal health data | Research |
| Andrews et al., | 2020 | Parents | 38 | Genetic conditions | US | Qualitative | Electronic health record | Research |
| Atkin et al., | 2021 | Patients, carers and/or members of the public | 42 patients & carers, 350 total study sample |  | UK | Mixed methods (workshops and survey) | Personal health data | Research |
| Avram et al., | 2018 | Patients | 590 | Cardiology | Canada | Survey | Electronic health record | Research |
| Bak et al., | 2021 | Patients and next of kin | 19 | Cardiac arrest | Netherlands | Qualitative | Personal health data, genetic and socio-economic data | Research |
| Beckjord et al., | 2011 | Patients and/or public | 16085 | Cancer | US | Survey | Electronic health record | Multiple stakeholders (inc. health care providers and research) |
| Bell et al., | 2014 | Public | 70 |  | US | Mixed methods (interview and survey) | Electronic health record | Research |
| Bernaerdt et al., | 2021 | Patients | 14 | "Vulnerable patients” | Belgium | Qualitative | Electronic health record | Multiple stakeholders (inc. health care providers and third parties including public and private research institutions/facilities, pharmaceutical companies, insurers and employers) |
| Bietz et al., | 2016 | Patients and/or public | 465 |  | US | Mixed methods (interview and survey) | Personal health data | Multiple stakeholders (inc. research and third parties) |
| Bond et al., | 2013 | Patients and/or public | 26 |  | UK | Qualitative | Online health discussion boards | Research |
| Bosisio et al., | 2021 | Patients | 22 |  | Switzerland | Qualitative | Bio samples, genomic and personal health data | Research |
| Bouras et al., | 2020 | Patients | 622 |  | US | Survey | Personal health data | Research |
| Brall et al., | 2021 | Public | 5516 |  | Switzerland | Survey | Bio samples, genomic and personal health data | Research |
| Burstein et al., | 2014 | Patients and parents | 309 |  | US | Mixed methods (interview and survey) | Genomic data | Research |
| Caine et al., | 2015 | Patients | 30 |  | US | Qualitative | Electronic health record | Health care providers |
| Carson et al., | 2019 | Patients | 60 | Fertility | UK | Mixed methods (interview and survey) | Personal health data | Research |
| Cherif et al., | 2021 | Patients | 674 |  | France | Survey | Personal health data | Health care providers |
| Cheung et al., | 2016 | Patients and/or public | 18 |  | US | Qualitative | Personal health data | Research |
| Coathup et al. | 2016 | Patients and/or carers | 40 | Myotonic Dystrophy patients | Japan | Survey | Personal health data | Research |
| Cochran et al., | 2015 | Patients and/or public | 67 |  | US | Qualitative | Personal health data | Health care providers |
| Courbier et al., | 2019 | Patients and/or carers | 2013 | Rare disease | International | Survey | Personal health data | Multiple stakeholders (inc. health care providers and research) |
| Crotty et al., | 2015 | Patients and/or carers | 53 | Older patients (75+) | US | Qualitative | Personal health data | Health care providers |
| Esmaeilzadeh & Mirzaei | 2018 | Patients and/or public | 1416 |  | US | Survey | Health information exchange | Health care providers |
| Esmaeilzadeh | 2019 | Patients | 493 |  | US | Survey | Health information exchange | Health care providers |
| Esmaeilzadeh | 2020 | Patients and/or public | 1624 |  | US | Survey | Health information exchange | Health care providers |
| Esmaeilzadeh et al., | 2021 | Public | 357 |  | US | Survey | Health information exchange | Health care providers |
| Esmaeilzadeh & Mirzaei | 2019 | Patients | 2013 |  | US | Experimental study | Health information exchange | Health care providers |
| Ford et al., | 2020 | Public | 18 |  | UK | Mixed methods (citizen’s jury and survey) | Electronic health record | Research |
| Franklin et al., | 2020 | Patients | 677 | Cancer | US | Survey | Electronic health record and mobile device data | Research |
| Frost et al., | 2014 | Patients | 115 | Cancer | Netherlands | Survey | Health information in online platforms | Research |
| Gaylin et al., | 2011 | Public | 1015 |  | US | Qualitative | Electronic health record | Health care providers |
| Grande et al., | 2013 | Patients and/or public | 3336 |  | US | Survey | Electronic health information | Multiple stakeholders (inc. care providers, commercial enterprises, public health departments) and uses (research, quality improvement, commercial marketing) |
| Grande et al., | 2014 | Patients and/or public | 3064 |  | US | Survey | Electronic health record | Multiple stakeholders (inc. marketing, research) |
| Grande et al., | 2015 | Patients and/or public | 3336* | Cancer | US | Survey | Personal health data | Multiple stakeholders (inc. health care providers and research) |
| Grande et al., | 2021 | Patients and/or public | 45 |  | US | Qualitative | Consumer digital data | Multiple stakeholders (inc. health care providers, insurance companies, researchers) |
| Goodman et al., | 2017 | Patients, carers and/or members of the public | 450 |  | US | Survey | Genomic data | Research |
| Grundstrom et al., | 2020 | Insurance customers | 452 |  | Finland | Survey | Personal health data | Insurance provider |
| Haddow et al., | 2011 | Patients and/or public | 19 |  | UK | Qualitative | Personal health data | Research |
| Harle et al., | 2018 | Patients | 32 |  | US | Qualitative | Electronic health record | Research |
| Harle et al., | 2019 | Patients | 734 |  | US | Survey | Electronic health record | Research |
| Hassan et al., | 2020 | Patients and/or public | 44 |  | UK | Qualitative | Genomic data | Multiple stakeholders (inc. health care providers and research) |
| Heath et al., | 2016 | Patients and/or public | 273 |  | US | Survey | Genetic data | Research |
| Helou et al., | 2021 | Patients and/or public | 112 |  | Japan | Survey | Personal health data | Research |
| Hentschel et al., | 2021 | Patients | 29 | Pregnant and breastfeeding mothers | US | Qualitative | Electronic health record | Research |
| Hiremath et al., | 2016 | Patients | 456 |  | US | Survey | Personal health data | Health care providers |
| Holm et al., | 2020 | Public | 994 |  | Denmark | Survey | Personal health data | Multiple stakeholders (inc. health care providers and research) |
| Horgan et al., | 2019 | Patients, industry, health care professionals and researchers | 71 patient groups, 446 total study sample |  | Europe | Mixed methods (interview and survey) | Personal health data and genome-based data | Research |
| Howard et al., | 2017 | Patients | 7 | Asthma | UK | Mixed methods (interview and survey) | Monitoring data | Multiple stakeholders (inc. health care providers and parents) |
| Howe et al., | 2018 | Patients and/or public | 9 papers |  | International | Systematic review | Clinical trial or public health research study data | Secondary research use |
| Huberty et al., | 2020 | Patients | 82 | Cancer | US | Survey | App data | Health care providers |
| Hui et al., | 2016 | Employees | 15 |  | US | Qualitative | Health risk assessment | Research |
| Hui et al., | 2020 | Patients and/or public | 200 |  | Malaysia | Survey | Personal health data in mobile health applications | Research |
| Hutchings et al., | 2020 | Patients and/or public | 35 papers (studies included a total of 56,365 respondents) |  | International | Systematic review | Personal health data | Research |
| Hwang et al., | 2020 | Patients and/or public | 391 |  | Taiwan | Survey | Electronic health record | Health care providers |
| Hyde et al., | 2020 | Patients and/or public | 942 |  | US | Survey | Wearable data | Multiple stakeholders (including health care provider, wearable manufacturer, health insurance company, community group, such as a local hospital fitness program or public health agency) |
| Jagsi et al., | 2017 | Patients | 621 | Cancer | US | Survey | Deidentified medical records | Research |
| Jagsi et al., | 2019 | Patients | 217 | Cancer | US | Mixed methods (deliberation sessions and survey) | Deidentified medical records | Research |
| Jones et al., | 2020 | Patients | 217** | Cancer** | US | Qualitative | Electronic health record | Research |
| Kalkman et al., | 2019 | Patients and/or public | 27 papers |  | International | Narrative Review | Personal health data | Research |
| Kim et al., | 2015 | Public | 800 |  | US | Survey | Electronic health record | Multiple stakeholders (inc. health care providers and research) |
| Kim et al., | 2017 | Patients | 394 |  | US | Survey | Clinical data and bio-samples | Research |
| Kim et al., | 2017 | Public | 800*** |  | US | Survey | Electronic health record | Multiple stakeholders (inc. health care providers and research) |
| Kim et al., | 2019 | Patients | 1246 |  | US | Survey | Electronic health record and biospecimens | Research |
| Kim et al., | 2019 | Patients and/or public | 170 | Older adults | South Korea | Survey | Personal health data | Multiple stakeholders (inc. family, hospital, researcher, government agency, device developer/corporation, and insurance company) |
| Kimura et al., | 2014 | Public | 657 (n=200 US, n=457 Japan) |  | Japan & US | Survey | Electronic health record | Multiple stakeholders (inc. health care providers, research and private companies) |
| King et al., | 2012 | Public | 723 |  | Australia | Mixed methods (focus groups and survey) | Personal health data | Research |
| Klein et al., | 2017 | Patients | 620 | Veterans | US | Survey | Electronic health record | Health care providers |
| Krahe et al., | 2019 | Patients | 249 |  | Australia | Survey | Personal health data | Research |
| Lee et al., | 2020 | Patients | 7 patients, 21 total study sample |  | South Korea | Qualitative | Personal health data | Multiple stakeholders (inc. health care provider and research) |
| Litchman et al., | 2018 | Patients and/or carers | 39 | Diabetes | US | Qualitative | Monitoring data | Carers |
| Lounsbury et al., | 2021 | Public | 352 |  | UK | Qualitative | Personal health data | Health care providers |
| Lu et al., | 2020 | Patients and/or public | 26 |  | Canada | Qualitative | Personal health data | Third party |
| Lucero et al., | 2015 | Public | 31 |  | US | Qualitative | Personal health data | Research |
| Luchenski et al., | 2013 | Patients and/or public | 2857 |  | UK | Survey | Electronic health records | Multiple stakeholders (inc. health care providers and research) |
| Luo et al., | 2020 | Patients | 109 |  | US | Survey | Patient generated data | Health care providers |
| Lysaght et al., | 2020 | Patients and/or public | 62 |  | Singapore | Qualitative | Personal health data | Multiple stakeholders (inc. health care providers, policy makers and research) |
| Mahlmann et al., | 2018 | Patients | 40 | Older adults | Switzerland | Qualitative | Genetic data | Research |
| Maiorana et al., | 2012 | Patients | 549 |  | US | Survey | Personal health data | Multiple stakeholders (inc. health care providers, pharmacists, private health insurers, government health insurers and local health departments) |
| Mamo et al., | 2013 | Patients | 36 | HIV | US | Qualitative | Personal health data | Secondary use |
| Mansour | 2018 | Patients | 45 |  | Egypt | Survey | Personal health data | Health care providers |
| Mazor et al., | 2017 | Patients | 15 |  | US | Qualitative | Electronic health records | Research |
| McGuire et al., | 2011 | Patients | 323 |  | US | RCT | Genomic data | Research |
| Medford-Davis et al., | 2017 | Patients | 982 |  | US | Qualitative | Electronic health record | Health care providers |
| Middleton et al., | 2020 | Patients and/or public | 36268 |  | International | Survey | Genomic data | Multiple stakeholders inc. researchers, healthcare providers, governments, profit and/or non-profit organisations |
| Middleton et al., | 2020 | Patients and/or public | 36268**** |  | International | Survey | Genomic data and health data | Research |
| Middleton et al., (b) | 2020 | Patients and/or public | 8967 |  | US, UK, Canada & Australia | Survey | Genomic data | Research |
| Middleton et al., (b) | 2020 | Patients and/or public | 8967***** |  | US, UK, Canada & Australia | Survey | Genomic data and health data | Research |
| Milne et al., | 2021 | Patients and/or public | 36268**** |  | International | Survey | Genomic data | Research |
| Milne et al., | 2019 | Patients and/or public | 8967***** |  | US, UK, Canada & Australia | Survey | Genomic data | Research |
| Miyamoto et al., | 2016 | Patients and/or public | 30 |  | US | Qualitative | mHealth data | Health care providers |
| Moon | 2017 | Patients and/or public | 18 papers |  | International | Review | Personal health data | Multiple stakeholders (inc. health care providers and commercial entities) |
| Murphy et al., | 2011 | Public | 1575 |  | Ireland | Survey | Personal health data | Research |
| Morrison et al., | 2014 | Patients | 3 patients, 70 total study sample |  | UK | Qualitative | Electronic health record | Multiple stakeholders (inc. health care providers and research) |
| Navarro-Millán et al., | 2019 | Patients | 31 | Rheumatoid Arthritis Patients | US | Qualitative | Patient reported outcome data | Multiple stakeholders (inc. health care providers and peers) |
| Nicholas et al., | 2019 | Patients and/or public | 211 |  | US | Survey | Health sensor data collected from smartphones | Multiple stakeholders (inc. health care providers, electronic health record systems and family members) |
| O'Brien et al., | 2019 | Patients | 3516 |  | US | Survey | Personal health data | Research |
| Page et al., | 2016 | Patients | 211 |  | Canada | Survey | Personal health data and biospecimens | Research |
| Papoutsi et al., | 2015 | Patients and/or public | 2761 |  | UK | Mixed methods (focus groups and survey) | Electronic health record | Health care providers |
| Parkin & Paul | 2011 | Public | 9 |  | New Zealand | Citizens jury | Personal health data | Research |
| Patel et al., | 2011 | Public | 200 |  | US | Survey | Personal health data | Technology initiatives (private and commercial) |
| Patil et al., | 2016 | Public | 20882 | 27 EU member countries | Europe | Survey | Personal health data | Multiple stakeholders (inc. family, health care providers, health insurance companies, private sector pharmaceutical companies and research) |
| Pedersen et al., | 2015 | Patients and/or public | 1004 |  | Canada | Survey | Electronic health record | Health care providers |
| Platt & Kardia., | 2015 | Public | 447 |  | US | Survey | Electronic health record and biological samples | Multiple stakeholders (health care providers, insurance companies, public health departments and research) |
| Platt et al., | 2018 | Public | 1011 |  | US | Survey | Personal health data | Multiple stakeholders (inc. health care providers, public health departments, research and information brokers) |
| Platt et al., | 2019 | Public | 1011****** |  | US | Survey | Personal health data | Multiple stakeholders (inc. health care providers, public health departments, research and information brokers) |
| Platt et al., | 2019 | Public | 890 |  | US | Survey | Personal health data | Multiple stakeholders (inc. health care providers and research) |
| Riordan et al., | 2015 | Patients and/or public | 3157 |  | UK | Survey | Electronic health record | Multiple stake holders (inc. health providers, researchers, policy-makers) |
| Rivas Velarde et al | 2021 | Patients and/or public | 73 |  | Switzerland | Citizens forum | Personal health data | Research |
| Romero & Young | 2021 | Patients and/or public | 25 |  | US | Qualitative | App data | Research |
| Sanderson et al., | 2017 | Public | 13000 |  | US | Survey | Bio samples and personal health data | Research |
| Satinsky et al., | 2018 | Patients | 8 |  | UK | Qualitative | Personal health data | Research |
| Schairer et al., | 2019 | Patients and/or public | 108 |  | US | Qualitative | Personal health data | Health technologies |
| Shen et al., | 2019 | Patients and/or public | 59 papers |  | International | Systematic review | Health information exchange | Research |
| Shen et al., | 2019 | Patients | 14 |  | Canada | Qualitative | Health information exchange | Health care providers |
| Small et al., | 2021 | Patients and carers | 28 |  | Canada | Qualitative | Adverse drug events | Health care providers |
| Small et al., | 2017 | Patients | 20 |  | Canada | Qualitative | Adverse drug events | Multiple stakeholders (inc. health care providers and research) |
| Smith et al., | 2012 | Patients | 54 | HIV | US | Qualitative | Personal health data | Health care provider |
| Soni et al., | 2020 | Patients | 25 |  | US | Mixed methods (interview and card sorting) | Electronic health record | Multiple stakeholders (inc. health care providers and research |
| Spencer et al., | 2016 | Patients | 40 | Chronic rheumatic disease | UK | Qualitative | Personal health data | Research |
| Steele Gray et al., | 2014 | Patients | 14 | Complex chronic disease and disability | Canada | Qualitative | Personal health data | Health care providers |
| Stevenson | 2015 | Patients and staff | 50 patients, 7 staff |  | UK | Qualitative | Electronic health record | Research |
| Street et al., | 2021 | Patients and/or public | 39 |  | Australia | Citizens jury | Personal health data | Multiple stakeholders (inc. private industry and research) |
| Tosoni et al., | 2021 | Patients | 222 |  | Canada | Survey | Personal health data | Research |
| Trinidad et al., | 2020 | Public | 1841 |  | US | Survey | Personal health data | Commercial companies |
| Tully et al., | 2018 | Patients and/or public | 34 |  | UK | Citizens jury | Electronic health record | Research |
| Tully et al., | 2019 | Patients and/or public | 36 |  | UK | Citizens jury | Personal health data | Commercial research |
| Tully et al., | 2020 | Public | 1978 |  | Sweden & UK | Discrete choice experiment | Personal health data and resource use | Research |
| Vidgen et al., | 2020 | Public | 1494 |  | Australia | Survey | Genomic data | Research |
| Weidman et al., | 2019 | Patients and/or public | 524 |  | US | Survey | Genetic data | Multiple stakeholders (inc. governmental institutions and private corporations) |
| Weng et al., | 2019 | Public | 1508 |  | US | Survey | Electronic health record | Research |
| Wetzels et al., | 2018 | Patients | 23 | Patients diagnosed with coronary artery disease, heart failure, or hypertension | Netherlands | Qualitative | Personal health data and non-medical health data | Multiple stakeholders (inc. research, insurance companies, pharmaceutical industry) |
| Whiddett et al., | 2016 | Public | 2438 |  | New Zealand | Survey | Electronic health record | Multiple stakeholders (inc. health care providers, government agencies) |
|  |  | Total number of participants | 164478 |  |  |  |  |  |
|  |  | Total number of included papers | 173 papers |  |  |  |  |  |

*Same study population as Grande et al., 2013

** Same study population as Jagsi et al., 2019

*** Same study population as Kim et al., 2015

**** Same study population as Middleton et al., 2020

***** Same study population as Middleton et al., 2020b

****** Same study population as Platt et al., 2019
