# Supplementary material for: Patient and Public Willingness to Share Personal Health Data for Third-Party or Secondary Uses: Systematic Review
Source: J Med Internet Res. 2024 Mar 5;26:e50421. doi: 10.2196/50421 (PMC10951832; doi:10.2196/50421)
Supplement: Multimedia Appendix 3 [file jmir_v26i1e50421_app3.docx]

| **Year of publication** | **Frequency** |
| --- | --- |
| 2011 | 7 |
| 2012 | 3 |
| 2013 | 4 |
| 2014 | 7 |
| 2015 | 11 |
| 2016 | 12 |
| 2017 | 12 |
| 2018 | 11 |
| 2019 | 23 |
| 2020 | 26 |
| 2021 | 19 |
|  | 135 |

| **Perspective** | **Frequency** |
| --- | --- |
| Patients | 47 |
| Patients and/or public | 48 |
| Public | 26 |
| Patients and/or carers | 5 |
| Patients, carers and/or members of the public | 2 |
| Parents | 1 |
| Patients and next of kin | 1 |
| Patients and parents | 1 |
| Employees | 1 |
| Patients, industry, health care professionals and researchers | 1 |
| Insurance customers | 1 |
| Patients and staff | 1 |
|  | 135 |

| **Study location** |  |
| --- | --- |
| US | 64 |
| UK | 18 |
| International | 10 |
| Canada | 9 |
| Australia | 4 |
| Switzerland | 4 |
| Netherlands | 3 |
| US, UK, Canada and Australia | 3 |
| Japan | 2 |
| New Zealand | 2 |
| South Korea | 2 |
| Europe | 2 |
| Japan & US | 1 |
| Ghana | 1 |
| Finland | 1 |
| Malaysia | 1 |
| Taiwan | 1 |
| Belgium | 1 |
| France | 1 |
| Denmark | 1 |
| Singapore | 1 |
| Egypt | 1 |
| Ireland | 1 |
| Sweden and UK | 1 |
|  | 135 |

| **Study design** | Frequency |
| --- | --- |
| Survey | 68 |
| Qualitative | 41 |
| Mixed methods | 12 |
| Citizens jury/forum | 5 |
| Review | 6 |
| Experimental study | 1 |
| Discrete choice experiment | 1 |
| RCT | 1 |
|  | 135 |

| **Type of data** | **Frequency** |
| --- | --- |
| Personal health data | 52 |
| Electronic health record/information | 31 |
| Genomic data | 9 |
| Health information exchange | 7 |
| Genetic data | 3 |
| Adverse drug events | 2 |
| App data | 2 |
| Biosamples, genomic and personal health data | 2 |
| Deidentified medical records | 2 |
| Genomic data and health data | 2 |
| Monitoring data | 2 |
| Biosamples and personal health data | 1 |
| Clinical data and bio-samples | 1 |
| Clinical trial or public health research study data | 1 |
| Consumer digital data | 1 |
| Electronic health record and biological samples | 1 |
| Electronic health record and biospecimens | 1 |
| Electronic health record and mobile device data | 1 |
| Health information in online platforms | 1 |
| Health risk assessment | 1 |
| Health sensor data collected from smartphones | 1 |
| mHealth data | 1 |
| Online health discussion boards | 1 |
| Patient generated data | 1 |
| Patient reported outcome data | 1 |
| Personal health data and biospecimens | 1 |
| Personal health data and genome-based data | 1 |
| Personal health data and non-medical health data | 1 |
| Personal health data and resource use | 1 |
| Personal health data in mobile health applications | 1 |
| Personal health data, genetic and socio-economic data | 1 |
| Wearable data | 1 |
|  | 135 |

| **Purpose and/or intended user** | **Frequency** |
| --- | --- |
| Research | 61 |
| Multiple stakeholders | 38 |
| Health care providers | 27 |
| Commercial | 2 |
| Carers | 1 |
| Health technologies | 1 |
| Insurance providers | 1 |
| Secondary research use | 1 |
| secondary use | 1 |
| Technology initiatives (private and commercial) | 1 |
| Third party | 1 |
|  | 135 |
